# Supplementary material for: Sweep Dynamics (SD) plots: Computational identification of selective sweeps to monitor the adaptation of influenza A viruses
Source: Sci Rep. 2018 Jan 10;8:373. doi: 10.1038/s41598-017-18791-z (PMC5762865; doi:10.1038/s41598-017-18791-z)
Supplement: Supplementary file 1 — Supplementary Material [file 41598_2017_18791_MOESM1_ESM.pdf]

Sweep Dynamics (SD) plots: Computational identification of selective sweeps to monitor the adaptation of influenza A viruses

Thorsten R. Klingen[1 †], Susanne Reimering[1 †], Jens Loers[1], Kyra Mooren[1], Frank Klawonn[2,3], Thomas Krey[4,5], Gülsah Gabriel[6,7] and Alice C. McHardy[1,5 *]

[1]Department for Computational Biology of Infection Research, Helmholtz Center for Infection Research, Braunschweig, Germany
[2]Biostatistics Group, Helmholtz Center for Infection Research, Braunschweig, Germany
[3]Department of Computer Science, Ostfalia University of Applied Sciences, Wolfenbüttel, Germany
[4]Institute of Virology, Hannover Medical School, Hannover, Germany
[5]German Center for Infection Research (DZIF)
[6]Viral Zoonoses and Adaptation, Heinrich Pette Institute, Leibniz Institute for Experimental Virology, Hamburg, Germany
[7]University of Lübeck, Germany

*Correspondence to: Alice.McHardy@helmholtz-hzi.de
[†]Co-first authors.

Supplementary Table 1

		09N	09S	10N	10S	11N	11S	12N	12S	13N	13S	14N	14S	15N	15S
**PB2**	**V344M**	0	0.0067	0.0267	0.23	**0.6433**	0.59	0.9267	0.9167	0.98	1	0.9467	1	1	0.9767
	**I354L**	0	0	0.02	0.23	**0.64**	0.59	0.9267	0.9167	0.9767	1	0.9633	1	1	1
	**D195N**	0	0	0.0033	0.01	0.1	0.23	0.17	0.05	**0.96**	1	0.9633	1	1	1
	**R293K**	0	0	0.0033	0	0.09	0.2133	0.15	0.0267	**0.96**	1	0.9567	1	1	1
	**V731I**	0	0	0.0033	0	0.06	0.23	0.16	0.05	**0.93**	1	0.9633	1	1	1
	**M66I**	0	0	0	0	0	0	0.04	0.05	**0.86**	1	0.9533	1	1	1
	**R54K**	0	0	0	0	0	0	0.04	0.05	**0.84**	1	0.9567	1	1	1
	**R251K***	0	0	0	0	0	0	0	0	0.0367	**0.6567**	0.0233	0	0	0
	**A184T**	0	0	0	0	0	0	0	0	0	0	0.0233	0	**0.7633**	0.5333
**PB1**	**I397M**	0	0	0	0.1533	0.24	0.4667	**0.6433**	0.5833	0.83	0.9767	0.9633	1	0.9533	1
	**I435T***	0	0	0	0.0867	0.22	0.4667	**0.6133**	0.51	0.0833	0	0	0	0	0
	**I435T**	0	0	0	0	0	0	0.02	0.0733	**0.8467**	1	0.9567	1	1	1
	**G154D**	0	0	0	0	0	0	0.0067	0.0733	**0.84**	1	0.9633	1	0.99	1
	**K52R***	0	0	0	0	0	0	0	0	0.0133	**0.6733**	0.0167	0	0	0
	**R211K**	0	0	0	0	0	0	0	0	0	0	0	0	**0.5533**	0.4933
**PA**	**L581M**	0	**0.8933**	0.9933	1	1	1	0.9967	1	1	1	1	1	1	1
	**N321K**	0	0	0	0.23	**0.6733**	0.5833	0.8833	0.95	0.96	1	0.96	1	1	1
	**A343T**	0	0	0	0.1567	0.2033	0.47	**0.6733**	0.5533	0.8633	0.23	0.0067	0	0	0
	**V100I**	0	0	0	0	0	0.02	0.0433	0	0.0567	**0.77**	0.94	1	1	1
	**I330V**	0	0	0	0.0033	0.23	0.1	0.1833	0.3967	0.0967	**0.77**	0.9367	1	0.9267	1
	**K361R**	0	0	0	0	0	0	0	0	0.0433	**0.77**	0.9367	1	0.91	0.7933
	**R362K**	0	0	0	0	0	0	0	0	0.0433	**0.77**	0.9467	1	1	1
	**P560H***	0	0	0	0	0	0	0	0	0.0233	**0.6633**	0.0233	0	0	0
	**I387V***	0	0	0	0	0	0	0	0	0	0	0.2267	**0.7867**	0.04	0
	**S522G***	0	0	0	0	0	0	0	0	0	0	0.2467	**0.7867**	0.04	0
**HA1**	**S203T**	0	**0.7067**	0.9667	0.9833	1	1	0.9967	0.9933	0.9833	1	1	0.99	1	1

	**S185T**	0	0	0.0067	0.22	**0.62**	0.69	0.9133	0.97	0.9633	0.98	1	0.99	1	0.9733
	**S143G***	0	0	0.0033	0.03	0.1733	0.46	**0.7167**	0.62	0.12	0.0133	0	0	0	0
	**A197T***	0	0	0.0033	0.1	0.2367	0.4733	**0.71**	0.6333	0.13	0.0133	0	0	0	0
	**N260D***	0	0	0	0	0.01	0.0233	**0.63**	0.42	0.02	0	0	0	0	0
	**S69T***	0	0	0	0	0	0	**0.62**	0.42	0.02	0	0	0	0	0
	**D97N**	0	0.0033	0.0067	0.0733	0.47	0.34	0.26	0.3467	**0.8367**	0.9633	1	0.99	0.9933	1
	**V234I***	0	0	0	0	0	0	0	0.0167	**0.6967**	0.3867	0.01	0	0.02	0.0267
	**K283E***	0	0.0033	0.0033	0	0	0	0	0.0167	**0.6467**	0.3833	0.01	0	0.0233	0.0267
	**K163Q**	0	0	0	0	0	0	0	0	0.08	**0.5533**	0.9833	0.99	0.9767	0.89
	**K283E**	0	0	0	0	0	0.0067	0.0067	0.0567	0.06	**0.5467**	0.9867	0.96	0.9767	0.9733
	**A256T**	0	0	0	0	0	0	0	0.02	0.0867	**0.5433**	0.99	0.9733	0.9767	0.9733
**NP**	**I373T**	0	**0.9**	1	0.9933	1	1	0.9933	0.96	0.97	1	0.7533	1	1	0.9867
	**V100I**	0	**0.7967**	0.9733	0.9733	0.9933	1	0.9933	1	0.96	1	0.9867	1	1	1
	**S498N**	0	0	0	0	0.0033	0.0833	0.06	0.11	**0.8667**	0.36	0.9533	1	1	1
	**E454D***	0	0	0	0	0	0	0	0	0.0167	**0.64**	0.02	0	0	0
	**N377S***	0	0	0	0	0	0	0	0	0.0167	**0.6333**	0.02	0	0	0
	**A22T**	0	0	0	0	0	0	0	0	0	0	0.2767	**0.7667**	0.1967	0.4333
	**M105T**	0	0	0	0	0	0	0	0	0	0	0.2767	**0.7667**	0.1967	0.4333
	**V425I**	0	0	0	0	0	0	0	0	0.0233	0	0.2533	0	**0.8033**	0.5667
	**V444I**	0	0	0	0	0	0	0	0	0	0	0.0033	0	**0.8033**	0.5667
**NA**	**V106I**	0.3787	**0.82**	0.9633	0.99	0.9767	0.9567	0.87	0.7767	0.1133	0.0467	0.0033	0	0	0
	**N248D**	0.3787	**0.8067**	0.98	0.9767	0.9833	0.95	0.8767	0.7633	0.0967	0.0367	0.0033	0	0	0
	**N369K**	0	0.0033	0.0033	0.2267	**0.6633**	0.8767	0.92	0.9633	0.9633	1	0.99	1	1	1
	**V241I**	0	0	0	0.22	**0.6567**	0.8333	0.8267	0.75	0.1067	0.0467	0	0	0	0
	**N44S***	0	0	0	0.11	0.22	0.4567	**0.61**	0.1833	0.0333	0	0	0	0	0
	**G41R***	0	0	0	0	0.0067	0.0267	**0.5267**	0.1767	0.02	0	0	0	0	0
	**I106V**	0	0	0	0	0.0033	0.0267	0.1067	0.1867	**0.8633**	0.9467	0.9967	1	1	1
	**N44S**	0	0	0	0	0.0033	0.0133	0.1167	0.1767	**0.8433**	0.9533	0.9933	0.9267	1	1
	**V241I**	0	0	0	0	0.0033	0.0233	0.1167	0.2033	**0.8433**	0.95	0.9967	1	1	1
	**N248D**	0	0	0	0	0.0033	0	0.0933	0.16	**0.8433**	0.95	0.9967	0.99	0.99	1
	**N200S**	0	0	0	0	0.0033	0.0233	0.1067	0.2033	**0.8367**	0.9167	0.98	0.8967	1	1

	**I321V**	0	0	0	0	0	0	0.0233	0.0533	0.1	**0.59**	0.9233	0.93	1	1
	**I34V**	0	0	0	0	0	0	0	0	0.0133	0.2767	**0.9167**	0.8567	0.96	0.91
	**K432E**	0	0	0	0	0	0	0	0	0.0167	0.2767	**0.9067**	0.93	0.97	0.9567
	**L40I**	0	0	0	0	0	0	0	0	0.0067	0.0133	0.2333	0.4433	**0.97**	0.9567
	**N386K**	0	0	0	0	0	0	0	0	0	0	0.2033	0.4433	**0.97**	0.9567
	**I117M**	0	0	0	0	0	0	0	0	0	0	0.0067	0.0167	**0.5733**	0.47
**M1**	**V80I**	0	0.0267	0.03	0.1733	**0.6433**	0.4867	0.8033	0.6533	0.05	0	0	0	0	0
	**K230R**	0	0	0	0.0367	0.0033	0.01	0.0567	0.0767	**0.9**	1	0.9967	1	1	1
	**V80I**	0	0	0	0	0	0	0.01	0.0767	**0.7133**	0.97	0.7633	0.2433	0.1533	0.24
	**M192V**	0	0	0	0	0	0	0.01	0.0767	**0.7133**	0.98	0.7633	0.2433	0.1667	0.24
	**V80I**	0	0	0	0	0	0.01	0.0467	0	0.13	0.02	0.2333	**0.7567**	0.8333	0.76
	**M192V**	0	0	0	0	0	0	0	0	0.0033	0	0.2	**0.7567**	0.8333	0.76
**M2**	**S13N***	0	0.0033	0.0033	0.0033	0.0633	0.0167	**0.5767**	0.4933	0.0267	0	0	0	0	0
	**V21G***	0	0	0	0	0.01	0.1233	0.0733	0.0467	**0.7**	0.32	0.02	0	0.1033	0
	**D21G**	0	0	0	0	0	0	0	0	0.12	**0.65**	0.9067	0.78	0.1767	0.3433
	**G21V**	0	0	0	0	0	0	0	0	0	0	0.0633	0.22	**0.7033**	0.6467
**NS1**	**I123V**	0	**0.6233**	0.91	0.9133	0.98	1	0.9933	0.9667	0.9967	1	1	1	1	1
	**L90I**	0	0	0	0.1767	0.25	0.4233	**0.6567**	0.66	0.8867	1	1	1	1	1
	**N205S**	0	0	0	0	0.0033	0.0167	0.0633	0.3067	**0.6133**	0.9733	0.9733	1	1	1
	**T80A***	0	0	0	0	0	0	0	0	0.0167	**0.6567**	0.0067	0	0	0
	**E55K**	0	0	0	0	0	0	0	0	0.02	0.0933	**0.61**	0.78	1	1
	**K131E**	0	0	0	0	0	0	0	0	0.0233	0.0933	**0.61**	0.78	1	1
**NS2**	**T48A**	0	0	0	0	0.0167	0.1233	0.1367	0.4033	**0.8267**	0.98	0.9767	1	1	1
	**N29S**	0	0	0	0	0	0	0	0	0.0167	**0.6867**	0.3667	0.76	0.93	0.9533

Table S1: Frequencies for ten pandemic H1N1 influenza A (pH1N1) proteins.

This table summarizes the raw frequency data for sweep-related changes in the pH1N1 proteins PB2, PB1, PA, HA, NP, NA, M1, M2, NS1 and NS2. The frequencies are further visualized in Figure 2 (A) – (K). Bold frequencies indicate a sweep-related change. Substitutions are marked with an asterisk if they quickly decrease in frequency in the seasons after their emergence, which indicates a potential hitchhiker.

Supplementary Table 2

		99N	99S	00N	00S	01N	01S	02N	02S	03N	03S	04N	04S	05N	05S	06N	06S	07N	07S	08N	08S	09N	09S	10N	10S	11N	11S	12N	12S	13N	13S	14N	14S	15N	15S
**HA**	**G5V***	0	0	**0.828**	0.284	0.264	0.152	0	0	0	0	0	0	0	0	0	0	0	0	0	0	0	0	0	0	0	0	0	0	0	0	0	0	0	0
	**K92T***	0	0	**0.828**	0.284	0.264	0.152	0	0	0	0	0	0	0	0	0	0	0	0	0	0	0	0	0	0	0	0	0	0	0	0	0	0	0	0
	**D271N***	0	0	**0.824**	0.284	0.264	0.152	0	0	0	0	0	0	0	0	0	0	0	0	0	0	0	0	0	0	0	0	0	0	0	0	0	0	0	0
	**Q33H***	0	0	**0.808**	0.284	0.092	0	0	0	0	0	0	0	0	0	0	0	0	0	0	0	0	0	0	0	0	0	0	0	0	0	0	0	0	0
	**R50G***	0	0	0	**0.6**	0.58	0.236	0	0	0	0	0	0	0	0	0	0	0	0	0	0	0	0	0	0	0	0	0	0	0	0	0	0	0	0
	**S199P***	0	0	0	**0.6**	0.58	0.236	0	0	0	0	0	0	0	0	0	0	0	0	0	0	0	0	0	0	0	0	0	0	0	0	0	0	0	0
	**P273S***	0	0	0.084	**0.6**	0.58	0.248	0	0	0	0	0	0	0	0	0	0	0	0	0	0	0	0	0	0	0	0	0	0	0	0	0	0	0	0
	**S247C***	0	0	0	**0.58**	0.58	0.236	0	0	0	0	0	0	0	0	0	0	0	0	0	0	0	0	0	0	0	0	0	0	0	0	0	0	0	0
	**A106V***	0	0	0	0	0.092	0.312	**0.94**	0.888	0.232	0.02	0	0	0	0	0	0	0	0	0	0	0	0	0	0	0	0	0	0	0	0	0	0	0	0
	**N144D***	0	0	0	0	0	0.28	**0.928**	0.888	0.108	0.02	0	0	0	0	0	0	0	0	0	0	0	0	0	0	0	0	0	0	0	0	0	0	0	0
	**S186G***	0	0.072	0	0	0.092	0.312	**0.924**	0.888	0.232	0.02	0	0	0	0	0	0	0	0	0	0	0	0	0	0	0	0	0	0	0	0	0	0	0	0
	**R50G**	0	0	0.008	0.096	0.064	0.288	0.052	0.112	**0.768**	0.904	0.956	1	0.976	0.968	0.5	0.16	0.28	0.056	0.004	0	0	0	0	0.008	0	0	0	0	0	0	0	0	0	0
	**E83K**	0	0	0	0	0	0.048	0.044	0.112	**0.768**	0.98	1	1	1	0.992	0.96	1	1	0.972	0.82	0.944	1	1	1	1	1	0.992	0.976	0.984	0.996	0.984	0.988	0.976	0.98	0.996
	**A131T**	0	0	0	0	0	0	0.024	0.072	**0.768**	0.98	1	1	1	1	1	1	1	1	1	1	1	1	1	1	1	1	0.976	0.992	1	1	0.996	0.996	1	0.992
	**S186G**	0	0	0	0	0	0	0.032	0.112	**0.768**	0.98	1	1	1	1	1	1	0.988	1	0.972	1	0.984	1	1	1	1	1	0.976	0.976	0.992	0.992	0.968	0.996	1	1
	**V202I**	0	0	0	0	0	0.048	0.044	0.112	**0.768**	0.98	1	1	1	1	1	1	1	1	1	1	0.992	1	1	1	1	1	0.976	1	1	0.992	0.996	0.996	0.992	1
	**W222R**	0	0	0	0	0	0.048	0.044	0.112	**0.768**	0.98	1	1	1	1	1	1	1	1	1	1	1	1	1	1	1	1	0.976	1	1	1	0.996	0.996	1	1
	**G225D**	0	0	0.008	0.096	0.064	0.288	0.052	0.112	**0.768**	0.98	1	1	0.96	0.436	0.084	0	0.012	0	0	0	0	0	0	0.008	0	0	0	0	0	0	0	0	0	0
	**L25I**	0	0	0	0	0	0	0	0.048	**0.744**	0.98	1	0.936	0.992	0.952	0.996	1	1	1	0.984	1	1	0.992	0.744	0.992	0.984	1	0.972	0.992	1	1	0.984	0.996	1	0.98
	**H75Q**	0	0	0	0	0	0	0	0.048	**0.744**	0.98	0.988	1	1	1	1	1	1	0.992	1	1	1	0.992	0.964	1	1	1	0.976	1	1	0.992	0.988	0.996	1	1
	**H155T**	0	0	0	0	0	0	0	0.048	**0.744**	0.98	1	1	1	1	1	1	1	1	1	1	1	1	1	1	1	1	0.976	1	1	1	0.996	0.996	1	1
	**Q156H**	0	0	0	0	0	0	0	0	**0.612**	0.98	1	1	1	1	0.968	1	0.988	1	0.992	1	1	1	1	1	1	1	0.976	0.992	1	1	0.996	0.996	1	1
	**N126D***	0	0	0	0	0	0	0	0	0	**0.756**	0.624	0.008	0.004	0	0	0	0	0	0	0	0	0	0	0	0	0	0	0	0	0	0	0	0	0
	**Y105H***	0	0	0	0	0	0	0	0	0	**0.68**	0.008	0	0	0	0	0	0	0	0	0	0	0	0	0	0	0	0	0	0	0	0	0	0	0
	**Y159F**	0	0	0	0	0	0	0	0	0	0.052	0.184	**0.976**	0.984	1	1	1	1	1	0.988	1	1	0.992	1	0.992	1	1	0.976	0.992	1	1	0.744	0.26	0.132	0.012
	**S189N**	0	0	0	0	0	0	0	0	0	0.104	0.264	**0.976**	0.984	1	1	1	1	1	1	1	0.96	0.188	0.024	0.008	0	0	0	0	0	0	0	0	0	0
	**K145N**	0	0	0	0	0	0	0	0	0	0	0.004	**0.876**	0.968	0.62	0.992	1	0.984	1	1	1	0.972	1	1	1	0.976	0.368	0.244	0.36	0.104	0.004	0	0	0	0
	**S227P**	0	0	0	0	0	0	0	0	0	0.132	0.2	**0.764**	0.94	1	1	1	1	1	1	1	1	1	1	1	1	1	0.976	1	1	1	0.996	0.996	1	1
	**V226I**	0	0	0	0	0	0	0	0	0	0	0	**0.664**	0.904	1	1	1	1	1	1	1	1	1	1	1	1	1	0.976	1	1	1	0.996	0.996	1	1
	**S193F**	0	0	0	0	0	0	0	0	0	0	0	0	0.04	**0.564**	0.916	1	0.988	1	1	1	1	1	1	0.992	1	1	0.976	1	1	1	0.988	0.988	1	1
	**D225N**	0	0	0	0	0	0	0	0	0	0	0	0	0.04	**0.564**	0.856	0.84	0.96	1	0.964	0.756	0.86	0.992	1	0.96	0.996	0.992	0.944	0.96	0.988	0.992	0.684	0.208	0.132	0.012
	**G50E***	0	0	0	0	0	0	0	0	0	0	0	0	0.024	0.032	0.492	**0.84**	0.012	0	0	0	0	0	0	0	0	0	0	0	0	0	0	0	0	0
	**G50E**	0	0	0	0	0	0	0	0	0	0	0	0	0	0	0.008	0	**0.62**	0.944	0.996	1	1	1	1	0.98	0.996	0.996	0.976	1	1	0.992	0.996	0.996	1	1

**K140I**	0	0	0	0	0	0	0	0	0	0	0	0	0	0	0.008	0	**0.62**	0.944	0.988	1	0.948	0.988	1	0.98	0.976	0.992	0.932	0.948	1	0.984	0.996	0.996	1	1
**Q261R**	0	0	0	0	0	0	0	0	0	0	0	0	0	0	0.008	0	**0.58**	0.912	0.984	0.964	0.984	0.94	0.848	0.96	0.988	0.972	0.976	1	1	1	0.964	0.98	0.904	0.884
**K173Q**	0	0	0	0	0	0	0	0	0	0	0	0	0	0	0	0	0	0.044	**0.524**	0.816	0.948	1	1	0.992	1	1	0.956	1	1	1	0.996	0.996	1	1
**E62K***	0	0	0	0	0	0	0	0	0	0	0	0	0	0	0	0	0	0	0	0	0.052	**0.7**	0.576	0.052	0.044	0.004	0	0	0	0	0	0	0	0
**K158N***	0	0	0	0	0	0	0	0	0	0	0	0	0	0	0	0	0	0	0	0	0.052	**0.7**	0.576	0.052	0.044	0.004	0	0	0	0	0	0	0	0
**N189K***	0	0	0	0	0	0	0	0	0	0	0	0	0	0	0	0	0	0	0	0	0.04	**0.7**	0.576	0.052	0.044	0.004	0	0	0	0	0	0	0	0
**N144K***	0	0	0	0	0	0	0	0	0	0	0	0	0	0	0	0	0	0	0	0	0.04	**0.696**	0.576	0.052	0.044	0.004	0	0	0	0	0	0	0	0
**V213A***	0	0	0	0	0	0	0	0	0	0	0	0	0	0	0	0	0	0	0	0	0.008	**0.564**	0.128	0.016	0.032	0	0	0	0	0	0	0	0	0
**P194L***	0	0	0	0	0	0	0	0	0	0	0	0	0	0	0	0	0	0	0	0	0.008	**0.56**	0.128	0.016	0.032	0	0	0	0	0	0	0	0	0
**K158N**	0	0	0	0	0	0	0	0	0	0	0	0	0	0	0	0	0	0	0	0	0	0.112	0.4	**0.94**	0.956	0.996	0.976	1	1	1	0.944	0.996	1	1
**N189K**	0	0	0	0	0	0	0	0	0	0	0	0	0	0	0	0	0	0	0	0	0	0.112	0.4	**0.94**	0.956	0.996	0.976	1	1	1	0.996	0.996	1	1
**T212A**	0	0	0	0	0	0	0	0	0	0	0	0	0	0	0	0	0	0	0	0	0	0.112	0.356	**0.86**	0.596	0.936	0.832	0.784	0.964	1	0.996	0.996	1	1
**N45S**	0	0	0	0	0	0	0	0	0	0	0	0	0	0	0	0	0	0	0	0	0	0.112	0.4	**0.852**	0.8	0.876	0.732	0.504	0.104	0.016	0	0.008	0	0
**D53N***	0	0	0	0	0	0	0	0	0	0	0	0	0	0	0	0	0	0	0	0	0	0	0.132	0.352	**0.668**	0.356	0.236	0.348	0.096	0.004	0	0	0	0
**I230V***	0	0	0	0	0	0	0	0	0	0	0	0	0	0	0	0	0	0	0	0	0	0	0.092	0.352	**0.668**	0.356	0.236	0.328	0.096	0.004	0	0	0	0
**Y94H***	0	0	0	0	0	0	0	0	0	0	0	0	0	0	0	0	0	0	0	0	0	0	0.044	0.352	**0.664**	0.356	0.236	0.348	0.096	0.004	0	0	0	0
**E280A***	0	0	0	0	0	0	0	0	0	0	0	0	0	0	0	0	0	0	0	0	0	0	0.144	0.344	**0.62**	0.356	0.236	0.348	0.096	0.004	0	0	0	0
**N312S**	0	0	0	0	0	0	0	0	0	0	0	0	0	0	0	0	0	0	0	0	0	0.012	0.204	0.48	0.152	**0.64**	0.672	0.6	0.884	0.98	0.988	0.988	1	1
**V223I**	0	0	0	0	0	0	0	0	0	0	0	0	0	0	0	0	0	0	0	0	0	0	0	0	0.016	**0.632**	0.732	0.632	0.896	0.996	0.996	0.996	1	1
**A198S**	0	0	0	0	0	0	0	0	0	0	0	0	0	0	0	0	0	0	0	0	0	0	0	0	0.02	**0.624**	0.672	0.608	0.884	0.988	0.988	0.988	0.988	0.992
**N145S***	0	0	0	0	0	0	0	0	0	0	0	0	0	0	0	0	0	0	0	0	0	0	0	0	0.004	**0.572**	0.556	0.292	0.012	0.012	0	0.008	0	0
**S45N**	0	0	0	0	0	0	0	0	0	0	0	0	0	0	0	0	0	0	0	0	0	0	0	0	0.008	0.02	0.164	0.348	**0.884**	0.984	0.996	0.98	1	1
**T48I**	0	0	0	0	0	0	0	0	0	0	0	0	0	0	0	0	0	0	0	0	0	0	0	0	0	0.02	0.164	0.348	**0.884**	0.984	0.996	0.988	1	1
**N278K**	0	0	0	0	0	0	0	0	0	0	0	0	0	0	0	0	0	0	0	0	0	0	0	0	0.008	0.028	0.164	0.348	**0.884**	0.996	0.936	0.996	1	1
**S157L**	0	0	0	0	0	0	0	0	0	0	0	0	0	0	0	0	0	0	0	0	0	0	0	0	0	0.02	0.164	0.316	**0.872**	0.936	0.84	0.864	0.872	0.988
**N145S**	0	0	0	0	0	0	0	0	0	0	0	0	0	0	0	0	0	0	0	0	0	0	0	0	0	0	0.004	0.12	**0.836**	0.984	0.94	0.972	1	0.992
**Q33R**	0	0	0	0	0	0	0	0	0	0	0	0	0	0	0	0	0	0	0	0	0	0	0	0	0	0.02	0.156	0.336	**0.808**	0.932	0.888	0.976	1	1
**N53D***	0	0	0	0	0	0	0	0	0	0	0	0	0	0	0	0	0	0	0	0	0	0	0	0	0	0	0.004	0.096	**0.52**	0.72	0.596	0.448	0.176	0.012
**T128A***	0	0	0	0	0	0	0	0	0	0	0	0	0	0	0	0	0	0	0	0	0	0	0	0	0	0	0.004	0.02	0.344	**0.7**	0.604	0.44	0.184	0.02
**R142G***	0	0	0	0	0	0	0	0	0	0	0	0	0	0	0	0	0	0	0	0	0	0	0	0	0	0	0.004	0.02	0.344	**0.7**	0.624	0.448	0.184	0.02
**N225D**	0	0	0	0	0	0	0	0	0	0	0	0	0	0	0	0	0	0	0	0	0	0	0	0	0	0	0	0	0	0	0.18	**0.512**	0.816	0.98
**Q311H**	0	0	0	0	0	0	0	0	0	0	0	0	0	0	0	0	0	0	0	0	0	0	0	0	0	0	0	0	0	0	0.176	**0.512**	0.816	0.98
**L3I**	0	0	0	0	0	0	0	0	0	0	0	0	0	0	0	0	0	0	0	0	0	0	0	0	0	0	0	0	0	0	0.176	0.488	**0.816**	0.98
**N144S**	0	0	0	0	0	0	0	0	0	0	0	0	0	0	0	0	0	0	0	0	0	0	0	0	0	0	0	0	0	0	0.176	0.492	**0.816**	0.98
**F159Y**	0	0	0	0	0	0	0	0	0	0	0	0	0	0	0	0	0	0	0	0	0	0	0	0	0	0	0	0	0	0	0.112	0.42	**0.816**	0.98
**K160T**	0	0	0	0	0	0	0	0	0	0	0	0	0	0	0	0	0	0	0	0	0	0	0	0	0	0	0	0	0	0	0.164	0.42	**0.772**	0.928

Table S2: Frequencies for the seasonal H3N2 influenza A (sH3N2) HA protein.

This table summarizes the raw frequency data for sweep-related changes in the sH3N2 HA protein. The frequencies are further visualized in Figure 3. Bold frequencies indicate a sweep-related change. Substitutions are marked with an asterisk if they quickly decrease in frequency in the seasons after their emergence, which indicates a potential hitchhiker.

Supplementary Table 3

<table>
<tr><th></th><th>Different substitution</th><th colspan="2">Identical substitution</th></tr>
<tr><th></th><th></th><th>Different season</th><th>Same season</th></tr>
<tr><td>Sampled data</td><td>S203T (2009S), S69T (2012N), K283E (2013S)</td><td>K163Q (2013S), A256T (2013S)</td><td rowspan="2">S185T (2011N), S143G (2012N), A197T (2012N), N260D (2012N), D97N (2013N), V234I (2013N), K283E (2013N)</td></tr>
<tr><td>Unsampled data</td><td>D97N (2012S), R133T (2013N), S185T (2013N), I321V (2013N)</td><td>K163Q (2014N), A256T (2014N)</td></tr>
</table>

Table S3: Comparison of detected sweep-related changes in the pH1N1 HA protein in sampled and unsampled data.

Similarities and differences between sweep-related changes in the pH1N1 HA protein in sampled data (300 sequences per season) downloaded from the NCBI database and unsampled data from GISAID's EpiFlu database.

Supplementary Table 4

<table>
<tr><th></th><th rowspan="2">Different substitution</th><th colspan="2">Identical substitution</th></tr>
<tr><th></th><th>Different season</th><th>Same season</th></tr>
<tr><td>Sampled data</td><td>G50E (2006S), G50E (2007N), K140I (2007N), K173Q (2008N), V213A (2009S), P194L (2009S), N145S (2011S), S157L (2013N), N53D (2013N)</td><td>Q261R (2007N), N45S (2010S), N225D (2014S), Q311H (2014S)</td><td rowspan="2">G5V (2000N), K92T (2000N), Q33R (2000N), D271N (2000N), R50G (2000S), S199P (2000S), P273S (2000S), S247C (2000S), A106V (2002N), N144D (2002N), S186G (2002N), R50G (2003N), E83K (2003N), A131T (2003N), V202I (2003N), W222R (2003N), G225D (2003N), L25I (2003N), H75Q (2003N), H155T (2003N), S186G (2003N), Q156H (2003N), N126D (2003S), Y105H (2003S), Y159F (2004S), S189N (2004S), S227P (2004S), K145N (2004S), V226I (2004S), S193F (2005S), D225N (2005S), N144K (2009S), K158N (2009S), N189K (2009S), E62K (2009S), K158N (2010S), N189K (2010S), T212A (2010S), D53N (2011N), Y94H (2011N), I230V (2011N), E280A (2011N), V223I (2011S), N321S (2011S), A198S (2011S), S45N (2013N), T48I (2013N), N278K (2013N), N145S (2013N), Q33R (2013N), T128A (2013S), R142G (2013S), L3I (2015N), N144S (2015N), F159Y (2015N), K160T (2015N)</td></tr>
<tr><td>Unsampled data</td><td>H311Q (2013N), D225N (2013N), G142R (2015N)</td><td>Q261R (2007S), N45S (2011N), N225D (2015N), Q311H (2015N)</td></tr>
</table>

Table S4: Comparison of detected sweep-related changes in the sH3N2 HA protein in sampled and unsampled data.

Similarities and differences between sweep-related changes in the sH3N2 HA protein in sampled data (250 sequences per season) downloaded from the NCBI database and unsampled data from GISAID's EpiFlu database.

Supplementary Figure 5

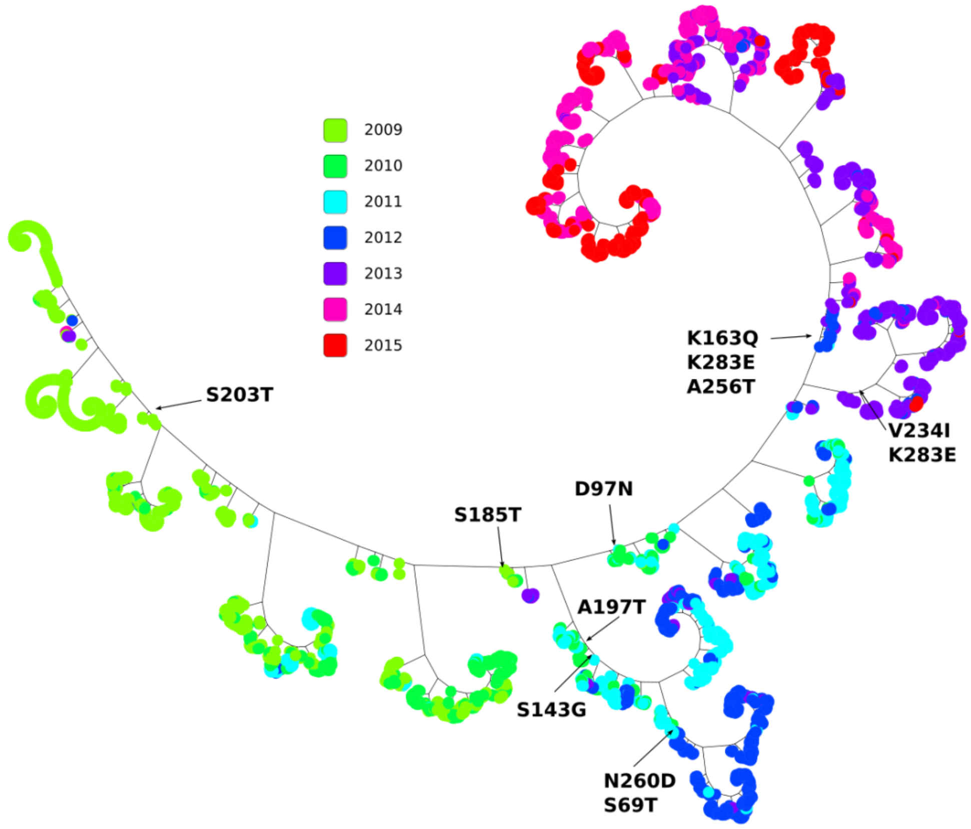

Figure S5: Phylogeny of the pH1N1 HA protein.

Phylogeny inferred on HA sequences showing the evolution of pH1N1 viruses from 2009N until the 2015S. Sweep-related substitutions are mapped onto the tree with the majority located on the main trunk of the tree.

Supplementary Figure 6

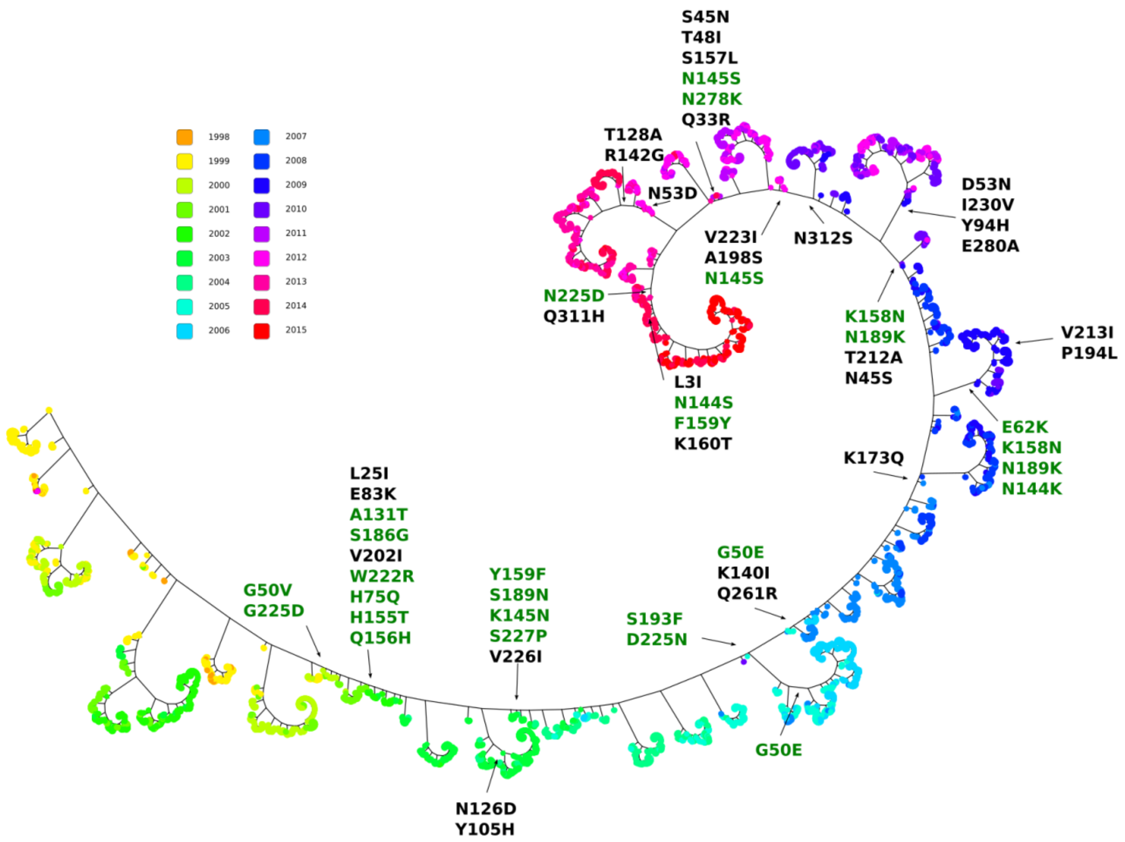

Figure S6: Phylogeny of the sH3N2 HA protein.

Phylogeny inferred on HA sequences showing the evolution of sH3N2 viruses from 1999N until the 2015S. Sweep-related substitutions are mapped onto the tree with the majority located on the main trunk of the tree. Substitutions that led to a new vaccine recommendation are colored in dark green.

Supplementary Table 7

PB2	substitution	season	support
	R251K	2013S	0.971
	R54K	2013N	0.95
	M66I	2013N	0.95
	D195N	2013N	0.939
	A184T	2015N	0.849
	V344M	2011N	0.749
	I354L	*2011N*	*0.424*
	R293K	*2013N*	*0.375*
	V731I	*2013N*	*0.088*

PB1	substitution	season	support
	K52R	2013S	0.977
	I435T	2013N	0.948
	R211K	2015N	0.859
	I397M	2012N	0.84
	G154D	2013N	0.8
	I435T	*2012N*	*0.199*

PA	substitution	season	support
	R362K	2013S	0.983
	K361R	2013S	0.983
	P560H	2013S	0.953
	I387V	2014S	0.911
	S522G	2014S	0.906
	N321K	2011N	0.893
	V100I	2013S	0.872
	I330V	2013S	0.863
	A343T	2012N	0.819
	L581M	*2009S*	*0.405*

HA	substitution	season	support
	K283E	2013N	0.926
	A197T	2012N	0.897
	S185T	2011N	0.896
	N260D	2012N	0.879
	K163Q	2013S	0.87
	S69T	2012N	0.84
	K283E	2013S	0.837
	A256T	2013S	0.835
	D97N	2013N	0.816
	V234I	2013N	0.788
	S203T	*2009S*	*0.39*
	S143G	*2012N*	*0.191*

**NP**	substitution	season	support
	N377S	2013S	0.98
	E454D	2013S	0.98
	M105T	2014S	0.909
	A22T	2014S	0.909
	V425I	2015N	0.878
	V444I	2015N	0.845
	V100I	2009S	0.834
	S498N	2013N	0.787
	I373T	2009S	0.781

**NA**	substitution	season	support
	N386K	2015N	0.908
	V241I	2013N	0.905
	N248D	2013N	0.905
	N369K	2011N	0.896
	G41R	2012N	0.892
	V241I	2011N	0.866
	I34V	2014N	0.846
	I117M	2015N	0.828
	N44S	2013N	0.801
	V106I	2009S	0.8
	I321V	2013S	0.798
	N44S	2012N	0.797
	N200S	2013N	0.787
	I106V	2013N	0.744
	N248D	2009S	0.733
	K432E	2014N	0.715
	L40I	*2015N*	*0.515*

**M1**	substitution	season	support
	V80I	2014S	1
	K230R	2013N	0.92
	V80I	2013N	0.913
	M192V	2013N	0.844
	V80I	2011N	0.73
	M192V	*2014S*	*0.428*

**M2**	substitution	season	support
	V21G	2013N	1
	D21G	2013S	1
	G21V	2015N	0.912
	S13N	2012N	0.821

**NS1**	substitution	season	support
	I123V	2009S	1

K131E	2014N	0.935
T80A	2013S	0.892
E55K	2014N	0.875
L90I	2012N	0.843
N205S	*2013N*	*0.026*

**NS2**	substitution	season	support
	T48A	2013N	1
	N29S	2013S	0.641

**HA (H3N2)**	substitution	season	support
	K145N	2004S	1
	W222R	2003N	0.999
	V202I	2003N	0.999
	E83K	2003N	0.999
	L25I	2003N	0.998
	H75Q	2003N	0.998
	H155T	2003N	0.998
	A106V	2002N	0.996
	S247C	2000S	0.995
	S199P	2000S	0.995
	R50G	2000S	0.995
	K140I	2007N	0.99
	Q311H	2014S	0.972
	N144S	2015N	0.972
	L3I	2015N	0.972
	K160T	2015N	0.972
	Q33H	2000N	0.952
	K92T	2000N	0.952
	G5V	2000N	0.952
	T128A	2013S	0.951
	R142G	2013S	0.951
	S193F	2005S	0.951
	D225N	2005S	0.951
	D53N	2011N	0.944
	E280A	2011N	0.943
	S45N	2013N	0.94
	N189K	2010S	0.936
	K158N	2010S	0.936
	R50G	2003N	0.926
	G225D	2003N	0.926
	N45S	2010S	0.924
	K173Q	2008N	0.923
	Y159F	2004S	0.921
	Q156H	2003N	0.918
	N126D	2003S	0.918
	S157L	2013N	0.908
	P273S	2000S	0.907
	F159Y	2015N	0.904

Y105H	2003S	0.903
N145S	2013N	0.892
V226I	2004S	0.881
N144K	2009S	0.88
V223I	2011S	0.876
N145S	2011S	0.876
N53D	2013N	0.869
P194L	2009S	0.867
N144D	2002N	0.845
A131T	2003N	0.833
S227P	2004S	0.829
D271N	2000N	0.816
G50E	2006S	0.816
N225D	2014S	0.803
I230V	2011S	0.801
S189N	2004S	0.799
N312S	2011S	0.795
S186G	2003N	0.793
Q261R	2007N	0.785
N278K	2013N	0.784
A198S	2011S	0.723
N189K	*2009S*	*0.592*
Y94H	*2011N*	*0.505*
T212A	*2010S*	*0.468*
T48I	*2013N*	*0.442*
Q33R	*2013N*	*0.442*
V213A	*2009S*	*0.411*
S186G	*2002N*	*0.398*
K158N	*2009S*	*0.378*
E62K	*2009S*	*0.378*
G50E	*2007N*	*0*

Table S7: Support values for each sweep-related substitution.

We catalogue sweep-related substitutions, their corresponding support value and the season in which they sweep. The branches in the phylogenetic tree on which sweep-related substitutions occurred are associated with support values calculated using the SH test. Substitutions having a support of less than 0.7, are written in italic.
